# A Health-Sector-Specific Occupational Health and Safety Management System Model

**DOI:** 10.3390/healthcare13030271

**Published:** 2025-01-30

**Authors:** Pınar Yeşilgöz, Kazım Yalçın Arga

**Affiliations:** 1 Occupational Safety Department, Institute of Pure and Applied Sciences, Marmara University, 34722 Istanbul, Turkey; 2 Occupational Health and Safety Department, Vocational School, İstanbul Esenyurt University, 34513 Istanbul, Turkey; 3 Department of Bioengineering, Faculty of Engineering, Marmara University, 34722 Istanbul, Turkey; kazim.arga@marmara.edu.tr

**Keywords:** multi-criteria decision-making, DEMATEL, AHP, management system, occupational health, occupational safety, healthcare worker

## Abstract

Background/Objectives: The health sector is one of the most important sectors, and occupational accidents and occupational diseases that health workers are exposed to are more important compared to those in other sectors. Especially, the increase in health and safety problems faced by hospital workers necessitates the development of an occupational health and safety (OHS) management system model specific to the health sector. Existing health and safety management systems generally do not sufficiently take sectoral dynamics into account, and the adaptation of these standards to the sectors is left to the individual efforts of the users. This situation leads to management systems that lack a sector-specific approach and do not adopt a common language and methodology. Methods: The aim of this study was to create an OHS management system specific to the health sector. While developing the model, AHP and DEMATEL, which are multi-criteria decision-making methods that help people to make complex decisions, were used. Results: By applying these methods, important criteria for the proposed model were determined. The criteria, including “Improvement of the management system of the health institution”, “Determination of control measures”, “Assessment of risks specific to the health institution”, “Identifying hazards specific to the health institution” and “Determining its context”, have been determined as priority criteria and weighted using the AHP and DEMATEL methods. Conclusions: As a result of the findings of this research, designing a unique occupational health and safety (OHS) management system that takes into account the dynamics of the health sector will contribute to the prevention of occupational accidents and occupational diseases in the health sector.

## 1. Introduction

Occupational health and safety is an interdisciplinary field that aims to reduce risks in the workplace and provide a safe working environment for employees. The World Health Organization (WHO) and the International Labour Organization (ILO) recognize this as an important human rights requirement, as well as the prevention of occupational accidents and occupational diseases [1]. The health sector is critical to public welfare because mistakes in this area can threaten human life [2,3,4].

Health services working environments are complex, and in this respect, employees are more prone to health-related problems than employees working in other sectors [5]. Therefore, in the health sector, which is one of the sectors most affected by the inadequate implementation of OHS practices, health workers face many potential hazards in health institutions. Healthcare workers are at higher risk compared to workers in other sectors, especially in terms of biological risks (e.g., bloodborne infections such as hepatitis B and C), ergonomic problems (e.g., heavy lifting or working in difficult positions), chemical ex-posure (e.g., sterilization chemicals) and psychosocial stressors (e.g., violence or long working hours) [6]. According to NIOSH, there are 29 types of physical hazards, 25 types of chemical hazards, 24 types of biological hazards, 10 types of psychosocial hazards and 6 types of ergonomic hazards in hospitals [7]. These hazards cause an increase in occupational accidents, adversely affect the health and safety of both patients and healthcare workers and cause a decrease in productivity and work performance. Healthcare workers are exposed to various risks, including infections, direct exposure to pathogenic microorganisms, injuries from needles and other sharp devices, exposure to various chemicals such as disinfectants and drugs, latex allergy, radiation, musculoskeletal disorders, stress, violence, burnout and improper management of medical waste.

Risks of biological contamination, which are rarely seen in other sectors, are among the distinguishing features of the health sectorHealthcare workers face infectious diseases, accidents caused by electric shock or fire hazards and hazards caused by exposure to radiation or harmful chemicals that are very harmful to health. The most common occupational hazards faced by health workers are musculoskeletal disorders and psycho-social hazards such as violence, stress and emotional burnout [8].

The OHS management system aims to provide a framework for managing OHS risks and opportunities. The ISO 45001 standard [9] aims to create a healthy and safe working environment for employees by preventing work-related injuries and occupational diseases. It also provides a platform for continuous improvement of the organization’s OHS performance [10]. The ISO 45001 standard consists of a cycle of planning, implementation, control and prevention, which is a common element in all ISO management systems standards The PDCA (Plan–Do–Check–Act) cycle in ISO 45001 is used in the development of OHS objectives, assessment of OHS risks, monitoring of implemented OHS activities and continuous improvement of OHS performance [11].

Occupational health and safety management system applications have many benefits for organizations. Occupational health and safety is integrated into the organization’s management system. OHSMSs are effective in reducing the risk of accidents and reducing the costs that may occur related to this. As a result, it increases productivity and profitability. In addition, OHSMSs improve compliance with the legal obligations of the organization [12]. The occupational health and safety management system is an indicator of the importance that the organization attaches to its employees, and this is a positive indicator for both customers and current and potential employees.

There is strong evidence in the literature that health-sector-specific OHS practices are inadequate. For example, while the generic OHS standard ISO 45001 provides a broad framework for managing workplace risks, it loses effectiveness when not adapted to sectoral requirements [13]. The diversity and intensity of the risks to which healthcare workers have been exposed in recent years in Turkey reveals the necessity of a sector-specific management system [14].

The existing literature shows that general OHS systems applied in the health sector cannot fully respond to sectoral requirements. For example, while ISO 45001 provides a general framework for reducing workplace hazards, it is insufficient in specific areas such as biological and chemical risks specific to the healthcare sector [15]. In contrast, the development of sector-specific management systems is critical to protecting employee health and improving workplace productivity [16].

ISO 45001 can be applied to all organizations regardless of factors such as the field of activity, number of employees and size of the organization. A management system specific to the health sector can be utilized to create a more effective management structure by taking into account the specific risks, dynamics and needs of this sector. Before creating an occupational health and safety management system model specific to the health sector, it is important to know the factors that are important for the application of the ISO 45001 Occupational Health and Safety Management System to the health sector.

A mistake made in the health sector can directly affect the health and life of patients, not only employees. The fact that health institutions provide 24 h uninterrupted service increases the risk of fatigue and error by limiting the rest periods of employees. Work should be performed correctly on the first attempt as mistakes usually cannot be corrected or compensated later. Accidents or negligence in the health sector can affect the general health of the community as well as the employees.

Burnout and turnover rates of healthcare workers directly affect service quality and sustainability. A study conducted during the pandemic period showed that healthcare workers experienced burnout symptoms [17].

General standards such as ISO 45001 provide an important framework for covering key occupational health and safety requirements, but more detailed and targeted industry-specific standards may be required to fully address specific industry-specific risks, working conditions and legal requirements.

ISO 45001 can be applied to all organizations regardless of factors such as the field of activity, number of employees and size of the organization. A management system specific to the health sector can be employed to create a more effective management structure by taking into account the specific risks, dynamics and needs of this sector. Before creating an occupational health and safety management system model specific to the health sector, it is important to know the factors that are important for the application of the ISO 45001 Occupational Health and Safety Management System to the health sector.

## 2. Materials and Methods

To guide OHSMSs, international institutions and organizations have published standards such as ILO-OHS-2001 [18], BS 8800 [19], OHSAS 18001:2007 [20] and ISO 45001:2018 [10]. The OHSAS 18001 standard, which has significant worldwide recognition, has been replaced by ISO 45001, developed by the International Organization for Standardization (ISO) as of March 2018. ISO 45001 provides an international framework for occupational health and safety management systems that benefits from a more comprehensive and integrated process-oriented approach.

ISO 45001 is designed to be implemented by any business or organization, regardless of the size of the organization and/or the sector in which it operates, and can be combined with various OHS programs (e.g., worker welfare and health) [21].

The advantages of the new OHS management system according to ISO 45001:2018 are as follows:

− Elimination of health and safety risks;
− Reduction in the number of occupational accidents and occupational diseases;
− Optimization of occupational health and safety practices;
− Ensuring leadership and commitment to the OHS management system;
− Monitoring and measurement support the management of the health and safety management system by assessing its performance levels [9].

**Figure 1:**
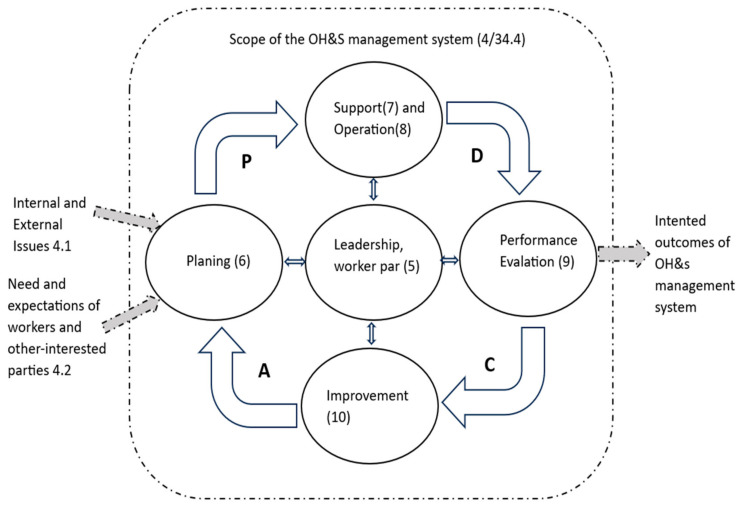
OHSMS and PDCA.

The Occupational Health and Safety management system is based on the Plan–Do–Check–Act (PDCA) concept shown in Figure 1 [22,23]. The PDCA concept is an iterative process used by the organization to achieve continuous improvement [24].

Plan: Identify and assess OHS risks and opportunities and other risks and opportunities; establish OHS objectives and processes necessary to achieve results based on the organization’s OHS policy.

Do: Implement the processes as planned.

Control: Monitor and measure activities and processes related to OHS policy and OHS objectives and reporting the results.

Take Action: Take measures to continuously improve OHS performance to achieve the intended results.

Implementing the ISO 45001 Occupational Health and Safety Management system demonstrates how committed an organization is to good working conditions, occupational health, welfare and equality in the workplace through strong leadership and employee engagement [9].

**Figure 2:**
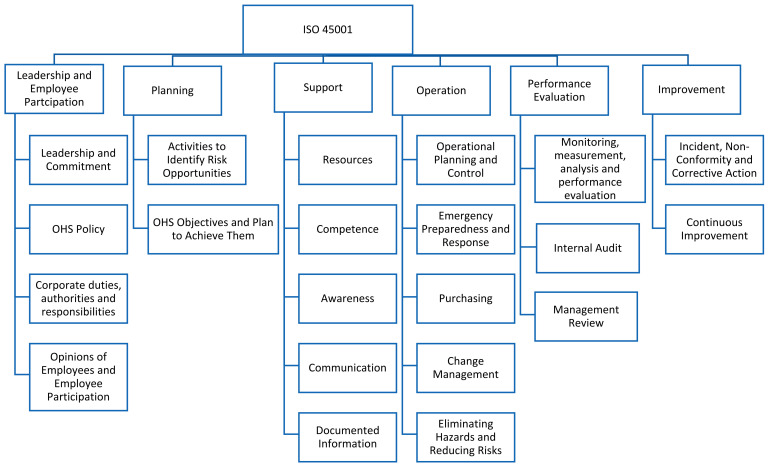
ISO 45001 Occupational Health and Safety Management System.

A management system model specific to the health sector requires knowledge of the sector’s important factors. The ISO 45001:2018 Occupational Health and Safety Management System provides a basis for the identification of critical factors in the occupational health and safety management system model specific to the health sector, as illustrated in Figure 2. The Analytic Hierarchy Process (AHP) and the Decision-Making Trial and Evaluation Laboratory (DEMATEL) methods are effective tools for multi-criteria decision-making processes suited to the complex structure of the healthcare sector. The AHP method enables the prioritization of factors that need to be addressed in the healthcare sector. Processes in the healthcare sector are tightly interconnected; for instance, the impact of a safety measure on employees can indirectly affect patient safety. DEMATEL facilitates the analysis of such interactions and dependencies, allowing the visualization of the network of relationships between factors. The analysis of cause-and-effect relationships among factors identifies the areas that require improvement [25].

One of the most important features of the DEMATEL method is that it presents a model that explains the relationships between criteria that determine the degree to which they influence each other and are affected by each other [26]. The DEMATEL method is used to determine the level of relationship between 8 main criteria and 27 sub-criteria in the study. The questionnaire prepared for the DEMATEL method application aims to determine the level of influence of the ISO 45001 Occupational Health and Safety Management System items on the decision-makers serving in the health sector.

DEMATEL (The Decision-Making Trial and Evaluation Laboratory) method was developed by the Battelle Memorial Institute in Geneva in the 1970s to produce solutions to complex problems. It is recognized as one of the best tools for determining the cause–effect relationship between evaluation criteria [27]. The DEMATEL method was developed to analyze and solve complex problems through group decision-making [28]. The DEMATEL method is a powerful method for collecting expert opinions and building a structural model [29].

As a method that examines the structure and relationships between variables and a valid number of decision alternatives, the DEMATEL method can prioritize the relationships between criteria based on the importance of their influence on each other. Criteria that have more influence on other criteria and have a high priority are referred to as influencing criteria, while criteria that are under more influence and are considered to have a low priority are referred to as affected criteria.

**Figure 3:**
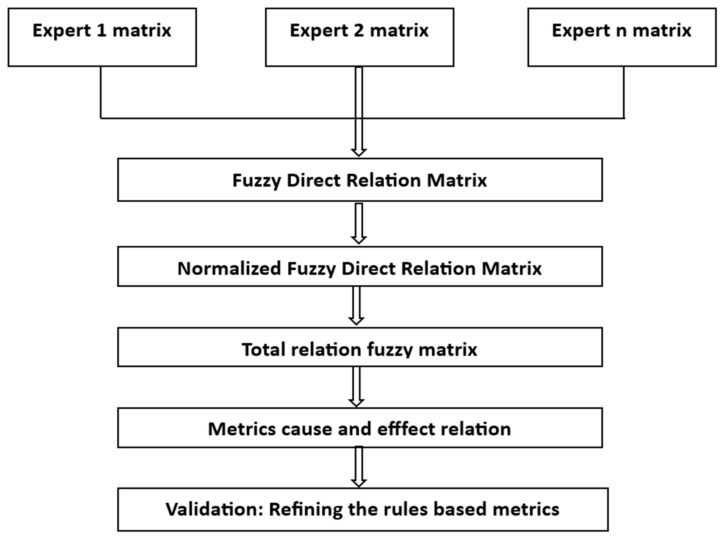
Fuzzy DEMATEL Method.

In the DEMATEL method, a pairwise comparison matrix similar to the Analytic Hierarchy Process (AHP) method is created. In the AHP method, pairwise comparisons are evaluated from equal importance to extreme importance between any two factors, whereas in the DEMATEL method, the direct effect ranging from no effect to high effect between any two factors is evaluated, as detailed in Figure 3, which outlines the steps of the DEMATEL method [30,31].

Phase 1: Creating the Direct Relationship Matrix

The first stage of the method is the construction of the direct relationship matrix. The values in the direct relationship matrix indicate the direct influence of variable *i* on variable *j*. It shows the direct relationship between variable *i* and variable *j*. The direct relationship matrix is shown in Equation (1).

(1) $$D=\left[\begin{array}{ccc} \begin{array}{c} \begin{array}{c} d_{11} \end{array}\\ \vdots \\ d_{i1} \end{array} & \begin{array}{c} d_{1j}\\ \vdots \\ d_{ij} \end{array} & \begin{array}{c} d_{1n}\\ \vdots \\ d_{in} \end{array}\\ \vdots & \vdots & \vdots \\ d_{n1} & \begin{array}{c} d_{nj} \end{array} & \begin{array}{c} d_{nn} \end{array} \end{array}\right]$$

When creating the direct relationship matrix, the numbers 0, 1, 2, 3 and 4 are used to indicate no impact, low impact, medium impact, high impact and very high impact respectively.

Phase 2: Normalization of the Direct Relationship Matrix

In the normalization stage, all values in the direct relationship matrix are normalized by dividing them by the largest value in the row and column sums. Equations (2) and (3) are used to normalize the direct relationship matrix.

(2) $$K=\frac{1}{max\sum_{j=1}^{n}a_{ij}}$$

*N* = *K* × *D* (3)

Phase 3: Calculation of the total relationship matrix

The normalized relationship matrix is transformed into the total influence matrix using Equation (4) and shown in Equation (5). The normalized direct relationship matrix is subtracted from the unit matrix, and its inverse is taken and then multiplied by itself again to obtain the total influence matrix.

(4) $$T=N\times \left(I-N{)}^{-1}\right.$$

Phase 4: Identification of Affecting and Affected Variables

The sum of rows is calculated by Equation (6) and the sum of columns by Equation (7).

(5) $$T=t_{ij,\;\;}\;i,\;j=1,\;2,\;\ldots n.$$

(6) $$\;D=\sum_{j=1}^{n}\begin{array}{c} T_{ij} \end{array}$$

(7) $$R=\overset{n}{\underset{j=1}{\sum }}t_{ij}$$

After calculating *D* and *R* values using Equations (6) and (7), *D* + *R* and *D* − *R* values are calculated. These values enable the determination of the relationships between the value and the variables and the grouping of variables as influencing/affected. A variable with a high *D* + *R* value is more related to other variables, while a variable with a low *D* + *R* value is less related to other variables. A variable with a positive *D* + *R* value is in the influencing group, while a variable with a negative *D* + *R* value is in the affected group.

Phase 5: Drawing the Influence Diagram and Relationship Map

The final stage of the method is to draw an influence diagram with the *D* + *R* and *D* − *R* values calculated from the total influence matrix and the threshold value determined. The threshold value is determined by the decision-makers within the expert team or, more commonly, by calculating the average values in the total relationship matrix. The size of the threshold value, whether large or small, can influence the interactions between the criteria in the decision-making process and affect the ease of finding a solution. When drawing the influence diagram, *D* + *R* values are placed on the horizontal axis of the coordinate plane, and *D* − *R* values are placed on the vertical axis.

AHP is mostly used in areas such as project evaluation and selection, diagnosis selection and ranking, treatment determination, organ transplantation, health service evaluation and policies [32].

Phase 1: Defining the Problem: The first step in the AHP method involves defining the problem clearly and establishing the decision hierarchy. A clear definition of the problem is important to extracting the criteria properly and to ensuring that there are no missing points.

Phase 2: Determination of Criteria and Alternatives: The second step of AHP is to determine the criteria and alternatives by utilizing the opinions of experts in the field, as indicated in Table 1, or by conducting a literature review on the subject.The hierarchical structure between the purpose of the problem, the criteria and the alternatives should be clearly and accurately established.

The AHP method was carried out by an expert panel (10 experts).

Phase 3: Creation of Pairwise Comparison Matrices: Pairwise comparison matrices use the AHP Pairwise Comparison Scale introduced by Saaty, as shown in Table 2.

Comparisons are based on pairwise comparisons of row elements to column elements.

Phase 4: The matrix is normalized by dividing the element in each column by the sum of the column in which it appears.

Phase 5: The “Priorities Vector” is obtained by averaging each row of the normalized matrix.

Phase 6: The Priorities Vector is multiplied by the initial pairwise comparison matrix to obtain the “All Priorities Matrix”.

Phase 7: The Coherence Index (*CI*) is calculated using Equation (8). *λ_max_* is equal to the average of the elements of a new matrix obtained by dividing each element of the entire Priorities Matrix by the elements of the Priorities Vector.

Phase 8: The “Consistency Ratio (*CR*)” is calculated using Equation (9).

(8) $$CI=\;\frac{\lambda_{max}-n}{n-1}$$

(9) $$CR=\frac{CI}{RI}$$

*RI* stands for the random value index. The consistency ratio is expected to be less than 0.1. If it is less than 0.10, the matrix is considered to be consistent; that is, the judgments of the decision-makers are consistent, as shown in the RI values in Table 3.

## 3. Results

The total impact matrix, created from the data collected with the DEMATEL questionnaire, is shown in Table 4.

(*D* + *R*) and (*D* − *R*) values calculated from the sum of rows and columns of the total effect matrix are shown in Table 5. *D_i_* (row totals) shows the total influence of each criterion on the other criteria. *R_j_* (column totals) shows how much each criterion is affected by other criteria. *D_i_* + *R_j_* expresses the total influence and impact of a criterion. A high value indicates that the criterion is important in the system. For *D_i_* − *R_j_*, a positive value indicates that the criterion is a cause (influencer), while a negative value indicates that it is a result (affected).

As shown in Table 5, the criterion with the highest *D_i_* + *R_j_* value is “Improving the management system of the health institution”, followed by “Identifying the hazards specific to the health institution” and “Determining the context of the health institution”.

As shown in Table 5, regarding the (*D* − *R*) values, “Determining the context of the health organization” is in the “affected” group as it is in the “affected” group and is driven by other criteria as an outcome criterion.

In Table 5, “Measuring OHS performance” and “OHS management of the health institution” are the strongest cause criteria with positive (*D* − *R*) values. These criteria have shown that they are the criteria that affect the other criteria the most.

**Figure 4:**
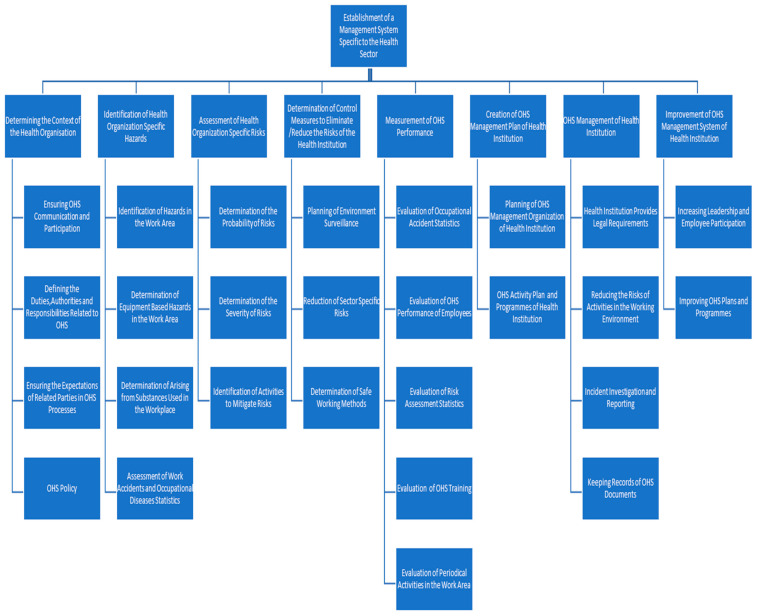
AHP hierarchical structure.

Table 6 presents the weighting of the main and sub-criteria derived from the application of the AHP method, as specified in Figure 4. The results indicate that the “Improvement of the management system of the health institution” is the most important criterion, ranking first. The second most important criterion is the “Determination of control measures for the health institution”, followed by the “Assessment of risks specific to the health institution”, which ranks third.

When examining the order of importance among the sub-criteria in Table 6, the criterion “Increasing leadership and employee involvement” emerges as the most significant. This criterion is followed by “Reducing sector-specific risks” and “Defining activities to reduce risks”, respectively.

## 4. Discussion

Since the activities in health services interact with each other, a disruption in one of the activities negatively affects patients/patient relatives and health workers. In this respect, effective health and safety management is needed to protect and improve occupational health and safety in health institutions and organizations. With effective health and safety management, holistic policies will be planned and implemented to ensure that employees work in a healthy and safe manner.

Since the ISO 45001 standard is a health and safety management system that is recommended to be applied regardless of the sector because of its high-level structure, the proposed management system model will respond to the needs for effective fulfillment of occupational health and safety activities by considering the specific characteristics of the health sector. The proposed management system can be used to determine the causes of occupational accidents occurring in the sector by ensuring the compliance of occupational safety regulations with technological developments in the health sector and to establish a system to prevent re-occurrence.

The occupational health and safety management system model proposed for implementation in the health sector is planned to meet the provisions of the occupational health and safety legislation; to be applicable, sustainable and easily understandable; and to emphasize participation. It should be ensured that the proposed system meets the legal requirements; is easily understandable for employers, employees and OHS professionals; and can be easily implemented in the health sector. The proposed model will be sustainable, modular and easily adaptable to changes in the size of the workplace, number of employees, etc., in the health sector.

An occupational health and management system model specific to the health sector and considering health and safety risks faced by health workers has been proposed. The level of healthcare services will be positively affected when healthcare workers feel safe. The improvements to be made as the implementation of the occupational health and safety management system model in the health sector will create significant results in both patient and employee safety. As a result, it will benefit occupational health and safety practices in the health sector.

Healthcare workers are exposed to risks such as infections, direct exposure to pathogenic microorganisms, injuries from needles and other sharp devices, exposure to various chemicals such as disinfectants and drugs, latex allergy, radiation, musculoskeletal disorders, stress, violence, burnout and mismanagement of medical waste. Identifying these health-organization-specific risks and control measures supports the implementation of effective risk assessment and control process in the proposed model. Identifying hazards specific to the health sector, assessing risks and taking necessary measures will ensure continuous improvement of OHS performance.

The OHS management of a health organization has a key role in the effective implementation of effective occupational health and safety management systems. It provides adequate support and resources for safety practices with the commitment and support of senior management [33].

“Improving the management system of the health institution” will contribute to a safer and healthier working environment in health institutions by increasing the effectiveness of occupational health and safety practices in the proposed model. Top management commitment and support, lack of employee participation in OHS practices, lack of communication on OHS issues and weak OHS attitudes can create problems in the implementation of the management system [34].

Inadequate commitment and support from senior management may result in employees giving less priority to the occupational health and safety management system and not actively participating in OHS practices [35].

A health and safety management system specific to the healthcare sector will ensure that occupational safety regulations align with contemporary technological advancements. Additionally, it will facilitate the identification of the causes of workplace accidents in the healthcare sector and establish preventive systems to avoid their recurrence. Undoubtedly, any investment or expenditure on such a system to prevent workplace accidents will be far less costly than the direct, indirect and external costs incurred after an accident occurs. To establish such a management system, it is essential to build a communication network among the government, workers and employers and to ensure active participation from all parties in occupational safety initiatives.

Ensuring active participation of employees in OHS practices is effective in identifying occupational safety problems and ensuring safe conditions [34]. To ensure the participation of employees, they should be provided with occupational safety training to increase the knowledge, competence and skills they need. Thus, regular training programs and awareness-raising campaigns to increase the level of knowledge and awareness of employees on OHS will encourage active participation in OHS practices. Positive changes in the attitudes and behaviors of employees towards safety with the training they receive will ensure the establishment of a strong OHS culture.

These and similar improvement activities in occupational health and safety must be sustainable. Putting all safety-related procedures in writing, participating in risk assessment and establishing legal regulations both within the workplace and within the legal regulations will contribute to sustainability [36].

It is important to determine appropriate indicators for measuring OHS performance and monitoring performance regularly. In the proposed model, indicators such as occupational accidents, risk assessment statistics, evaluation of OHS training and periodical activities in the work area reveal the weaknesses and strengths of the management system.

Taking into account the views and needs of relevant parties and the support and leadership of senior management is critical for the success of management systems. In this context, the fact that AHP and DEMATEL methods are based on expert opinions in the fields of health services and occupational safety increases the acceptability and success of the system. While the AHP method is used to determine the weights of the criteria in the model, it systematizes the decision-making process by dividing the problems into a hierarchical structure and contributes to the simplification of complex structures. The DEMATEL method, on the other hand, allows a more realistic and comprehensive evaluation by analyzing the causal relationships between the criteria. However, the fact that both methods work with a large number of criteria and sub-criteria may cause the process to be time-consuming, increase the error rate and also carry the risk of the results being based on subjective data since they are based on expert opinions.

### Practical Recommendations and Future Prospects

In order to implement an effective management system, the context in which the organization operates must be well understood and determined. The health institution should determine its internal and external factors, the needs and expectations of the relevant parties, its legal obligations and its strategic direction. Internal factors in determining the context of the healthcare organization can be the organizational structure of the organization, distribution of tasks and competence levels of employees, medical devices, equipment used and internal communication channels. External factors may be legal regulations specific to the health sector, expectations of patients and society from health services, technological developments and innovations in health services.

Small-scale pilot projects can be implemented to determine the effectiveness of the management system, and as a result, the system will be improved in line with the feedback received from the field. Creating common platforms that will increase communication between the state, employers, trade unions and health institutions can contribute to making occupational safety management systems in the health sector more effective, sustainable and innovative.

## 5. Conclusions

An occupational health and safety management system specific to health institutions can be created to meet the needs of employees in terms of health and safety in health institutions. With the model created in this way, it can be effective in facilitating operations, increasing service quality and preventing labor losses that may occur at the same time. It is also very important in terms of solving the problems experienced by health workers. Finding solutions to the health and safety problems of health workers can increase the quality of health services. The adaptation of the proposed model to health services can benefit health institutions serving worldwide.

Studies should be carried out to identify hazards for each unit in health institutions. Identification of hazards in each unit is important for controlling these risks. Comprehensive analysis of the risks and requirements specific to the health sector increases the success of the occupational health and safety management system. Occupational accidents and occupational diseases will decrease with a management system model focused on the risks to which healthcare workers are exposed. The management system for risk reduction will enable health institutions to provide services in a safer environment for both employees and patients. A well-designed management system will improve the quality and safety of healthcare services by reducing the risks faced by healthcare workers, patients and their relatives.

Performance indicators should be regularly monitored and evaluated to increase the effectiveness of an occupational health and safety management system specific to the health sector and ensure continuous improvement. Regular monitoring of performance indicators such as records of occupational accidents and diseases effectively improves health institutions’ functioning and creates a safe working environment by preventing occupational accidents, increasing the safety of employees and strengthening the safety culture.

Limited budget and personnel in health institutions may negatively affect the implementation process of the management system. This may make it difficult for the system to be fully effective in all areas. Since the health sector has to keep pace with rapidly developing technology, updating the management system and integrating it with new technologies can be time-consuming and costly. Constantly changing local and international laws and regulations in the health sector may make it difficult to adapt to the system. Therefore, continuous legal harmonization is required. Healthcare workers may need to be trained on the new management system, which requires time and resources. In addition, there may be resistance that affects the applicability of the system.

In the health sector, effective communication between different disciplines should be ensured, and coordination between all employees should be strengthened. Open communication between managers, employees and patients ensures the success of the system. In addition, since the security of health data is important, the management system should comply with data security standards, and the confidentiality of personal health information should be ensured.

To ensure the participation of employees, they should be provided with occupational safety training to increase the knowledge, competence and skills they need. Thus, regular training programs and awareness-raising campaigns to increase the level of knowledge and awareness of employees on OHS will encourage active participation in OHS practices. Positive changes in the attitudes and behaviors of employees towards safety with the training they receive will ensure the establishment of a strong OHS culture. These and similar improvement activities in occupational health and safety must be sustainable. Putting all safety-related procedures in writing, participating in risk assessment and establishing legal regulations both within the workplace and within the legal regulations will contribute to sustainability.

It is important to create a dynamic structure in accordance with the ever-changing technological and legal regulations of the occupational safety management system specific to the health sector. In the management system model, studies should be carried out to make it suitable for legal regulations in different countries. For this, general solutions will be developed by testing in different countries. In addition, the management system should be regularly harmonized with national and international standards. For this, studies should be carried out on how to integrate artificial intelligence and other technologies into occupational safety processes.

## Figures and Tables

**Table 1:** The characteristics of the experts.

Jobs	Experience Duration
Occupational safety experts specialized in the health sector	10 years
Academics in the field of health management	15 years
Academics in the field of occupational health and safety	10 years
Occupational health and safety managers	10 years
Health managers	15 years
Quality managers	20 years
Health professionals specialized in risk management	10 years

**Table 2:** AHP Likert scale.

Likert Scale	Options of Selection
1	equally important
3	moderately important
5	strongly important
7	very strongly important
9	extremely important
2, 4, 6, 8	values in the middle between two options

**Table 3:** Random value indices.

n	1	2	3	4	5	6	7	8	9	10	11	12	13	14	15
*RI*	0	0	0.58	0.89	1.12	1.24	1.32	1.41	1.45	1.49	1.51	1.48	1.56	1.57	1.59

**Table 4:** Total relationship matrix for main criteria (T).

	Determining the Context of the Health Organization	Identification of Hazards Specific to the Health Institution	Assessment of Risks Specific to the Health Institution	Determination of Control Measures of the Health Institution	Measuring OHS Performance	Establishment of the Management Plan of the Health İnstitution	OHS Management of the Health Organization	Improving the Management System of the Health Organization
Determining the context of the health organization	0.596	0.768	0.768	0.739	0.738	0.807	0.807	0.746
Identification of hazards specific to the health institution	0.777	0.818	0.972	0.938	0.894	0.882	0.882	0.852
Risks specific to the health institution evaluation	0.777	0.972	0.818	0.938	0.894	0.882	0.882	0.852
Determination of control measures of the health institution	0.777	0.972	0.972	0.784	0.894	0.882	0.882	0.852
Measuring OHS performance	0.878	1.081	1.081	1.009	0.882	1.035	1.035	1.001
Establishment of the management plan of the health institution	0.819	0.880	0.880	0.847	0.851	0.809	0.963	0.932
OHS management of the health organization	0.819	0.880	0.880	0.847	0.851	0.963	0.809	0.932
Improving the management system of the health organization	0.887	0.963	0.963	0.925	0.996	1.043	1.043	0.855

**Table 5:** Specification of main criteria weights by DEMATEL method.

	*D_i_*	*R_j_*	*D_i_* + *R_j_*	*D_i_* − *R_j_*
Determining the context of the health organization	5.972	6.334	12.307	−0.361
Identification of hazards specific to the health institution	7.019	5.737	12.757	1.281
Assessment of risks specific to the health institution	7.019	4.960	11.979	2.058
Determination of control measures of the health institution	7.019	4.183	11.202	2.835
Measuring OHS performance	8.005	3.405	11.411	4.599
Establishment of the management plan of the health institution	6.986	2.527	9.514	4.459
OHS management of the health organization	6.986	1.707	8.694	5.279
Improving the management system of the health organization	7.679	6.860	14.539	0.819

**Table 6:** Weighting of main and sub-criteria by AHP method.

Main Criteria	Weight	Sub Criteria	Weight
Determining the context of the health organization	0.01882	Ensuring OHS communication and participation	0.06048
		Defining the duties, authorities, and responsibilities related to OHS	0.08034
		Relevant parties in OHS processes ensuring expectations	0.54935
		OHS policy	0.30983
Identification of hazards specific to the healthcare organization	0.14737	Identification of hazards specific to the working area	0.46825
		Identification of equipment-related dangers in the working area	0.10249
		Determination of hazards arising from the substances used in the work area	0.14667
		Evaluation of occupational accident and disease statistics	0.28259
Assessment of risks specific to healthcare facilities	0.14723	Determination of the probabilities of risks	0.08096
		Determination of the severity of risks	0.18839
		Identification of actions to mitigate risks	0.73064
Determination of control measures of the health institution	0.15673	Planning of workplace surveillance	0.08096
		Reducing sector-specific risks	0.73064
		Safe working methods determination of the organization	0.18839
Measurement of occupational health and safety (OHS) performance	0.04459	Evaluation of occupational accident statistics	0.34727
		Evaluation of employees performance	0.08592
		Risk assessment statistics evaluation	0.39645
		Evaluation of OHS training	0.12507
		Evaluation of periodic activities in the workplace	0.04529
Establishment of the management plan of the health institution	0.06123	Planning of the occupational health and safety (OHS) management organization in healthcare facilities	0.75
		OHS activities of the health institutionplans and programs	0.25
OHS management in healthcare facilities	0.02854	Ensuring that the health institution meets the legal requirements	0.10207
		Reducing the risks of activities in the working environment	0.6291
		Accident investigation and reporting	0.22646
		Maintenance of records for OHS documents	0.04236
Improvement of the management system in healthcare facilities	0.39549	Increasing leadership and employee participation	0.75
		Improvement of OHS plans and programs	0.25

## Data Availability

Data are contained within the article.
